# Who is the person in person-centered care? Reflections from palliative care

**DOI:** 10.1017/S1478951526102314

**Published:** 2026-04-30

**Authors:** Bianca Sakamoto Ribeiro Paiva, William Breitbart

**Affiliations:** 1 GPQual – Research Group on Palliative Care and Quality of Life, Barretos Cancer Hospital, Barretos, Brazil; 2 Teaching and Research Institute, Barretos Cancer Hospital, Barretos, Brazil; 3 VAHLOR – Virtual Academy for Health Learning and Research, Kingston, Ontario, Canada; 4 Department of Psychiatry & Behavioral Sciences, Memorial Sloan Kettering Cancer Center, New York, NY, USA

In a hospital bed for more than 30 days, receiving palliative care, a patient with advanced cancer reported that her pain was “under control.” Clinical parameters were stable, and symptom management appeared adequate. Still, something remained unresolved. After a brief silence, a different concern emerged: it was not the pain itself, but uncertainty about who she had become in the context of illness. Her words did not describe a physical symptom, but a rupture in identity, the sense that the person she once was no longer fully accessible, accompanied by the fear of having lost herself.

This situation is recurrent in palliative care, and it is precisely here that we must understand the important distinction between treating the patient and caring for the person.

Person-centered care is widely recognized as a fundamental principle of contemporary medicine. It is frequently cited in clinical practice and institutional documents; however, its meaning is often not fully assumed and is, in some ways, relativized. After all, who is the “person” in person-centered care? We might further ask: which dimensions of the person become most relevant in the context of serious illness?

Eric Cassell proposes that suffering occurs when the integrity of the person is threatened – “Suffering is experienced by persons, not merely by bodies” (Cassell 1982). This formulation invites us to reconsider the focus of care beyond disease, directing it toward the preservation of the person as a whole. Disease affects organs and systems, but suffering affects the person, representing a complex integration of body, mind, identity, relationships, and meaning. As Cassell himself states, “the patient is not the disease” (Cassell 1982).

In this context, suffering may be understood as a threat not only to physical integrity, but to the coherence of the self, particularly when identity and meaning are disrupted.

Although disease can be described, measured, and classified, the experience of illness strongly resists such reduction. Patients do not suffer only from physiological changes, but because illness disrupts the continuity of their lives, their roles shift and identities are threatened, undermining what once provided direction and purpose. This leads us to an important reflection: in clinical practice, symptom control is not sufficient when a person’s sense of identity is threatened.

Cicely Saunders expanded this understanding through the concept of “total pain,” recognizing that suffering is multidimensional, encompassing physical, psychological, social, and spiritual dimensions (Saunders 1990). In other words, pain cannot be understood or treated in isolation from the person’s life context. Effective care requires attention to the person as a whole, not merely to the disease process (Saunders 1990).

In clinical practice, physical symptoms are profoundly influenced by emotional, relational, and existential factors. Fear of death, unresolved family conflicts, loss of autonomy, and anticipatory grief may intensify suffering in ways not captured by traditional clinical instruments and not always adequately addressed in routine clinical approaches. Conversely, when these dimensions are acknowledged, symptoms may become more manageable. Adequate symptom control requires an integrated approach that simultaneously considers the physical and psychosocial aspects of suffering (Bruera and Sweeney 2002).

Within this broader perspective, William Breitbart highlights the centrality of meaning in the human experience. In *Meaning-Centered Psychotherapy in the Cancer Setting*, Breitbart argues that the search for meaning is a fundamental human motivation, particularly in the face of suffering (Breitbart 2016). When illness threatens life, it also threatens the structures that sustain meaning, namely identity, purpose, and continuity of the self.

Individuals who once perceived themselves as active, independent, or productive may experience a profound disruption of identity. In this context, existential questions emerge: Why is this happening? What still matters? What remains meaningful? Biomedical interventions may not adequately address these questions, as they lie at the core of the illness experience.

Loss of meaning may manifest as hopelessness, demoralization, or emptiness. Thus, suffering may be understood not only as the presence of pain, but as the erosion of meaning. Person-centered care, therefore, requires attention to this dimension, not as a secondary aspect, but as a central element of care (Breitbart 2016).

The preservation of meaning is closely related to the concept of dignity. Harvey Max Chochinov describes dignity as deeply linked to a sense of identity, autonomy, and personal worth (Chochinov et al. 2002). Dignity may be understood as a way of being in the world, shaped by how individuals perceive themselves and are recognized by others, whereas meaning reflects the existential “why” that sustains one’s life. While dignity can be threatened or even eroded by illness and external circumstances, meaning may still be preserved or reconstructed. This distinction is illustrated in extreme human experiences, such as those described by Viktor Frankl, in which dignity may be stripped away, yet meaning can still be sustained through one’s attitude toward suffering. Preserving dignity goes beyond maintaining function; it involves sustaining continuity of identity as a person and meaning, even in the face of decline. Interventions that value life history, recognize personal values, and foster connections may help preserve this sense of continuity (Chochinov et al. 2002).

Meaning, in turn, reflects an existential capacity to make sense of one’s experience, including suffering itself. Dignity is sustained not only through clinical interventions, but through relational and existential recognition of the person (Chochinov et al. 2002).

Listening therefore assumes a central role, allowing access not only to symptoms but also to the meanings attributed to experience. In this sense, listening is not merely a clinical tool, but a relational act that acknowledges the person in their uniqueness.

Patients often seek not only answers, but recognition, the confirmation that their experience has been seen and understood. The opportunity to narrate one’s story may, in itself, alleviate suffering, even when the disease cannot be altered. The role of the clinician in person-centered care goes beyond the application of technical knowledge. It requires the ability to approach the person who is suffering, to tolerate uncertainty, and to remain present in situations that cannot be fully resolved. Relief of suffering requires understanding the person who suffers. This perspective suggests that person-centered care is not merely a model of care, but an ethical stance toward the human experience of illness.

Returning to the initial situation, the patient’s primary concern was not pain, but the loss of identity and meaning. Situations such as this reveal that the central goal of care is not only to relieve symptoms, but to address the deeper question of what it means to be a person in the context of illness.

To care for the person, ultimately, is to care for that which gives meaning to their life.
